# Experimental investigation on enhanced combustion of methanol/heavy fuel oil by droplet puffing at elevated temperatures

**DOI:** 10.1038/s41598-024-64482-x

**Published:** 2024-06-24

**Authors:** Xiaoyu Chen, Wuqiang Long, Yang Wang, Ge Xiao, Pengbo Dong, Zixin Wang, Xi Xi

**Affiliations:** School of Energy and Power Engineering, Dalian University of Technology, Dalian, 116024 China

**Keywords:** Methanol/heavy fuel oil blends, Puffing, Bubble rupture, Single droplet combustion, Ignition delay, Energy science and technology, Crude oil

## Abstract

To achieve high-efficiency combustion of heavy fuel oil (HFO), this study investigated the combustion characteristics of methanol/HFO droplets with methanol content from 10 to 30% using the suspension method under ambient temperature from 923 to 1023 K. The combustion of methanol/HFO droplets was summarized as a two-phase process consisting of six typical stages, emphasizing liquid phase. Especially, the fluctuation evaporation stage, induced by frequent and intense puffing, was identified as prominent character. Both the ignition delay and lifetime of HFO and methanol/HFO droplets decreased with increasing ambient temperatures. For the methanol/HFO droplet, the ignition delay and droplet lifetime increased with the increasing methanol content. Prominently, compared to HFO, HM10 had the most significant reduction in droplet lifetime and TINL under the same operating conditions, which indicated that the addition of 10% methanol accelerated the combustion process and reduced soot generation. Additionally, the thermos-dynamic characteristics of methanol/HFO droplets were investigated. Puffing was primarily attributed to superheating of methanol and pyrolysis of heavy components in HFO, which resulted in active and passive rupture of bubbles. Similarity and maximum deformation were employed to qualitatively distinguish between them. The obtained findings aimed to develop a promising alternative fuel to reduce emissions and preserve energy.

## Introduction

Shipping continues to be the primary mode of transportation for international trade, and heavy-duty and marine diesel engines play a vital role in this domain by providing high power output and improved fuel efficiency^1,2^. Marine diesel engines heavily rely on heavy fuel oil (HFO) as their main fuel source. Despite its relatively low cost, HFO is considered to be the lowest-grade fuel due to poor atomization caused by high viscosity. Additionally, HFO exhibits low ignitability and combustibility, resulting in suboptimal fuel economy and increased pollutant emissions from diesel engines^3^. Therefore, the utilization and development of HFO-based diesel engines face significant challenges in addressing environmental pollution and energy crisis concerns^4,5^.

To tackle these challenges, proposals for alternative fuels have emerged to encourage cleaner combustion and sustainable energy solutions. Notable examples encompass biodiesel^6^, alcohol-ether fuels^7^, ammonia^8,9^, and hydrogen^10^. Among these alternatives, methanol is regarded as a promising candidate due to its low carbon content, oxygen-rich nature, and advantageous characteristics for high-efficiency and clean combustion. Moreover, methanol remains in a liquid state under normal conditions, further enhancing its competitiveness. However, utilizing pure methanol for combustion in diesel engines may require modifications to the engine's injection system; otherwise, due to the low calorific value, high latent heat of vaporization, and strong corrosiveness of methanol, it can result in reduced thermal efficiency, unstable combustion, and potential corrosion of the fuel supply system.

To address these problems, researchers have proposed the blending of HFO with other substances as a means of improving the fuel's physical properties and its potential for secondary atomization resulting from micro-explosions^11–13^. Previous research has mainly focused on water/HFO blends due to their low cost and ease of production^12,14,15^. However, the addition of water to the fuel blend, while cost-effective, does not contribute to heat generation during combustion and hurts overall thermal efficiency^11^. Further studies indicate that methanol/HFO blended fuels demonstrate promising prospects for application. These blends consist of a complex mixture where each component has a different boiling point. The variation in boiling points among the components promotes bubble nucleation within droplets during evaporation under engine conditions, thereby enhancing atomization quality. Furthermore, the high viscosity and surface tension of HFO enables it to blend effectively with methanol, resulting in a methanol-in-oil structure. This can help compensate for methanol's corrosiveness and poor lubrication properties within the fuel supply system, eliminating the need for extensive modifications to the existing engine fuel systems.

Understanding the phenomenon of combustion is necessary for the improvement of blended fuels. Extensive studies have adopted a droplet combustion approach to explore the combustion characteristics of fuel droplets^16–23^, including phenomenological flame structure analysis and the variations in droplet temperature and diameter during combustion. In most cases, mono-component fuels exhibit stable flame structures with regular decreases in droplet diameter^18,24^. However, in the case of blended fuels such as 2,5-dimethylfuran (DMF)/biodiesel and Jatropha oil/DMF, the presence of components with lower boiling points can lead to preferential vaporization. This phenomenon can result in occurrences such as puffing or micro-explosions, which have been observed to cause significant deformations in the flame structure^16,17,20^. Additionally, puffing or micro-explosions have a notable impact on heat transfer and mass diffusion surrounding the droplet, leading to unique behaviors in droplet diameter and temperature during droplet combustion.

The combustion performance of blended fuel droplets has been the subject of numerous investigations. Wang et al.^20^ investigated the influence of DMF concentration on the combustion of Jatropha oil/DMF blended fuel droplets and divided the combustion process into four stages, including ignition delay, first micro-explosive combustion, *d*^2^ law combustion, and second micro-explosive combustion stage. The flame deformation resulting from micro-explosions can be categorized into four types and the impact of the DMF concentration on ignition delay and the burning rate was clarified. Another study by Won et al.^19^ studied the combustion process of water/n-decane blended droplets at elevated temperatures and varying water content. The results showed a decrease in ignition delay time with increasing ambient temperature. Interestingly, the average burning rate was found to be relatively insensitive to both ambient temperature and water volume ratios. Zhang et al.^25^ investigated the puffing, auto-ignition, and combustion characteristics of an n-pentanol/diesel droplet, revealing that higher concentrations of n-pentanol led to longer auto-ignition delays at 880 K but resulted in shorter delays at 930 K. Tao et al.^21^ studied the combustion characteristics of a droplet of lubricating oil and gasoline under the ambient temperatures (from 673 to 873 K) and gasoline blending ratios (from 0 to 20%). The results mainly emphasized the auto-ignition delay, droplet lifetime, and the occurrence time of puffing decreased with the increase of gasoline content. Recently, HFO-based blended fuels such as water/HFO^26^ and bio-oil/HFO^27^ have received significant attention. The combustion of HFO-based blended fuels has been found to result in lower soot generation and a higher burning rate.

As mentioned above, the blend of alternative fuels and HFO promotes the occurrence of micro-explosive combustion. Methanol is an ideal additive due to its advantageous properties such as low boiling point, low carbon content, simple molecular structure, high oxygen content, and close solubility to HFO. However, to the best of our knowledge, there is limited research on the combustion characteristics of methanol/HFO blends. The considerable difference in boiling points between methanol and HFO creates favorable conditions for bubble nucleation and growth in methanol/HFO droplets. Additionally, the high viscosity and surface tension of HFO, resulting from its heavy and polar components^28^, significantly influence droplet behavior. Therefore, studying the combustion characteristics of methanol/HFO droplets is crucial for expanding our understanding of blended fuel combustion.

In this study, the combustion characteristics of methanol/HFO droplets with a methanol content from 10 to 30% under ambient temperatures of 923, 973, and 1023 K were investigated using the suspended droplet method. The combustion process of methanol/HFO droplets was divided into six distinct stages, with particular emphasis on liquid-phase combustion. The effects of ambient temperature and methanol content on the evolution of normalized squared diameter and droplet temperature were studied. Detailed analyses were conducted on ignition delay, droplet lifetime, and TINL for both HFO and the blended fuel at elevated ambient temperatures. Additionally, thermos-dynamic characteristics of methanol/HFO droplets resulting from puffing were examined, and the phenomena of puffing induced by active and passive bubble rupture were clarified. The comprehensive findings from the present study on the combustion characteristics of methanol/HFO blended fuel were of significant importance for the understanding of the combustion process in real engines, and it also laid the foundation for understanding the mechanisms of chemical reaction kinetics in methanol blended fuels and exploring the physical and chemical nature of combustion.

## Experimental methods

### Preparation of fuels

In this study, three types of HFO and methanol blended fuels were prepared, with a methanol content ranging from 10 to 30. The methanol/HFO blend with 30% methanol content denoted HM30 (the letter “H” represents HFO (#180) while “M” denoted methanol, and “30” represented the concentration of methanol is 30%.). The methanol/HFO blends were prepared by mechanically mixing HFO (#180), methanol, and surfactants at a high stirring speed under fixed temperature conditions. The physical properties of HFO and methanol are presented in Table 1. The surfactants used were Span-80 (TCI Development Co., Ltd. HLB = 4.3), OP-10 (TCI Development Co., Ltd. HLB = 14.5), and Tween 80 (TCI Development Co., Ltd. HLB = 15), which were individually added to HFO and methanol. The total amount of surfactants added was 0.5% by weight of the HFO-methanol mixture. The methanol/HFO emulsified fuel prepared in this study can be stable for at least 2 weeks.

**Table 1:** Physical properties of HFO and methanol.

	HFO	Methanol
Flash point [K]	380	284
Cetane number	50	5.7
Latent heat of vaporization [kJ/kg]	254	1100
Boiling point [K]	896–1046	338
Density at 323 K [kg/m3]	982.25	790
Surface tension at 323 K [mN/m]	29	20.6
Kinematic viscosity at 323 K [mm^2^/s]	134.28	0.522
Lower heating value [MJ/kg]	41.727	19.7

The addition of methanol to HFO aims to enhance the combustion and emission performance of HFO. However, it is important to consider an upper limit concentration of methanol, as an excessive amount can result in a lower calorific value. In this study, a maximum methanol concentration of 30% was selected to investigate its effects on combustion characteristics due to excessive methanol content can not ensure sufficient fuel energy content. Experimental tests revealed that HFO droplets exhibited auto-ignition when exposed to ambient temperatures exceeding 873 K. Therefore, the ambient temperature range of 923–1023 K was chosen for the investigation, with a temperature interval of 50 K to capture significant changes in combustion behavior across the selected range.

### Experimental apparatus

**Figure 1:**
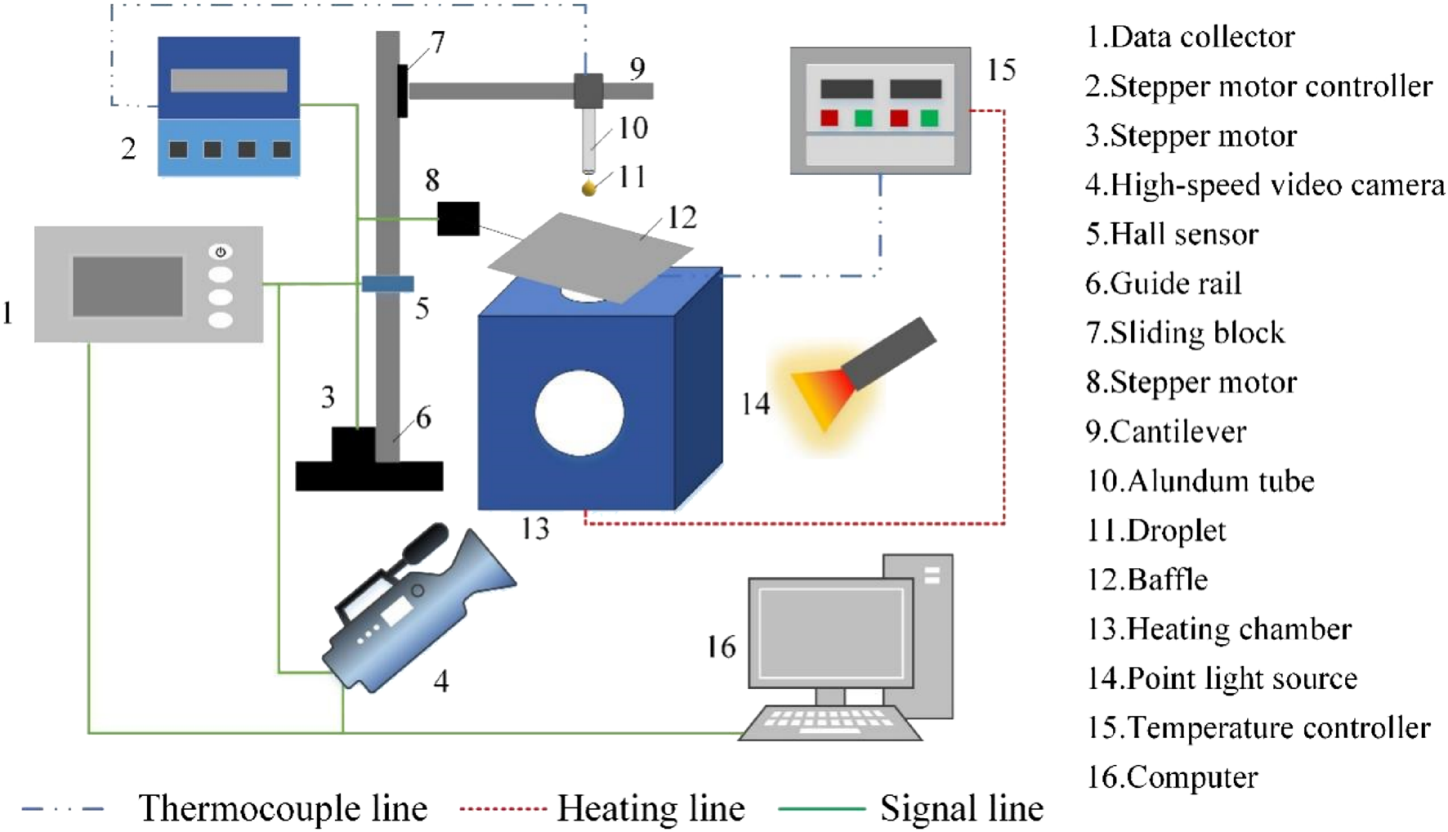
Schematic depicting the experimental apparatus.

In this study, the suspended droplet method was employed to investigate the combustion characteristics of methanol/HFO droplets. This method was chosen due to the rapid and frequent occurrence of puffing processes in methanol/HFO droplets, allowing for continuous observation of droplet shape and position during combustion. The experimental methodology primarily referred to previous research^25,29^. The experimental setup, as depicted in Fig. 1, consisted of three main systems: a heating system, a droplet production and transfer system, and a data acquisition system. Six heating rods, along with a PID feedback temperature controller and a cubic heating chamber measuring 150 mm on each side, formed the heating system. To provide insulation, the outer surface of the heating chamber was wrapped with glass fiber felt, while two quartz-glass windows with an 80 mm diameter were installed at the front and rear of the chamber for direct observation of droplet behavior. Six heating rods were placed around the heating chamber. The temperature of the heating chamber was measured by a K-type armored thermocouple, which was connected to the PID temperature controller, offering precise temperature control up to 1100 K with an accuracy of ± 1 K.

The droplet production and transfer system included a microliter syringe, guide rail, sliding block, thermocouple, baffle, and two electric motors. An alundum tube with a diameter of 4 mm was used to encase the thermocouple without insulation in order to secure it and shield it from the elevated environmental temperatures. To maintain a uniform initial temperature for the droplets, a baffle consisting of fiber paper and silicone resin was utilized, which had the ability to withstand temperatures up to 1100 K. The guide rail and sliding block, powered by a stepper motor and controlled by a stepper motor controller, facilitated the movement of the droplet into the heating chamber.

The data acquisition system simultaneously collected temperature data from the exposed thermocouple using a data collector with a sampling rate of 25 Hz, and high-speed image data with a resolution of 1280 × 1024 pixels using a high-speed digital camera. A point light source, in combination with micro-lens attached to the front of the high-speed digital camera, was used to provide backlighting for the scene. When the droplet reached its focal point, a hall sensor located on the support frame of the guide rail was triggered, sending a TTL signal to both the data collector and camera systems. This allowed for the simultaneous collection of temperature and image data. For specific device names and models, please refer to Ref.^30^.

### Processing and analysis of experimental data

Two main sources of natural flame light emission are chemiluminescence and soot incandescence, with the latter exhibiting much higher luminosity, serving as an indicator of soot production during combustion. The brightness of natural flame light emission which is correlated linearly with the soot measurement, has been widely adopted to characterize the amount of soot generation^31–33^. Thus, in this study, the method of using the brightness of natural flame light emission to characterize the amount of soot generation was adopted.

The spatially integrating natural luminosity (SINL), a key parameter for quantifying flame brightness, was determined by analyzing high-speed camera images using MATLAB. The methodology involved extracting the R, G, and B chromatic components of each pixel within the flame region and weighting these components to calculate the brightness value of that pixel. By integrating the brightness values of all pixels within the flame region, the SINL value of the flame was obtained. Consistent shooting conditions, including shooting distance and exposure, were maintained throughout the experiment to minimize experimental errors, and multiple repetitions were conducted for each operational point. Additionally, integrating SINL over time provided TINL, which reflected the overall amount of soot generated during the entire combustion process^34^.

To investigate the combustion characteristics of methanol/HFO droplets, the variations in droplet temperature and size were measured simultaneously at specified experimental conditions. To ensure the accuracy of the results, each condition was tested three times and averaged values were recorded and presented in the following discussion. For more information on the image processing method and the analysis of experimental uncertainties, please refer to Ref.^30,35^.

## Results and discussion

### The combustion process of methanol/HFO droplet

**Figure 2:**
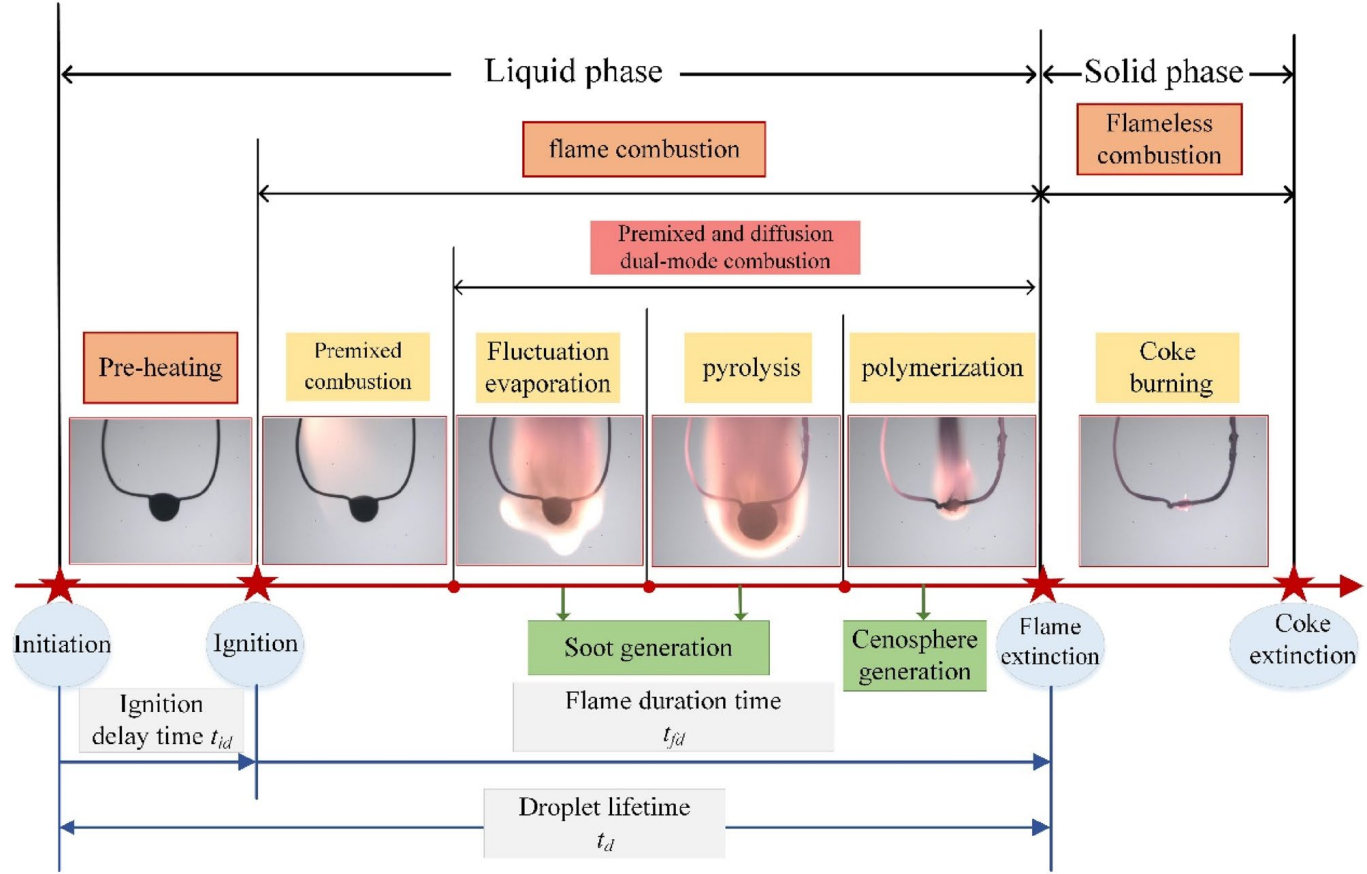
The diagram of combustion stages of methanol/HFO droplet with the illustration of high-speed images.

**Figure 3:**
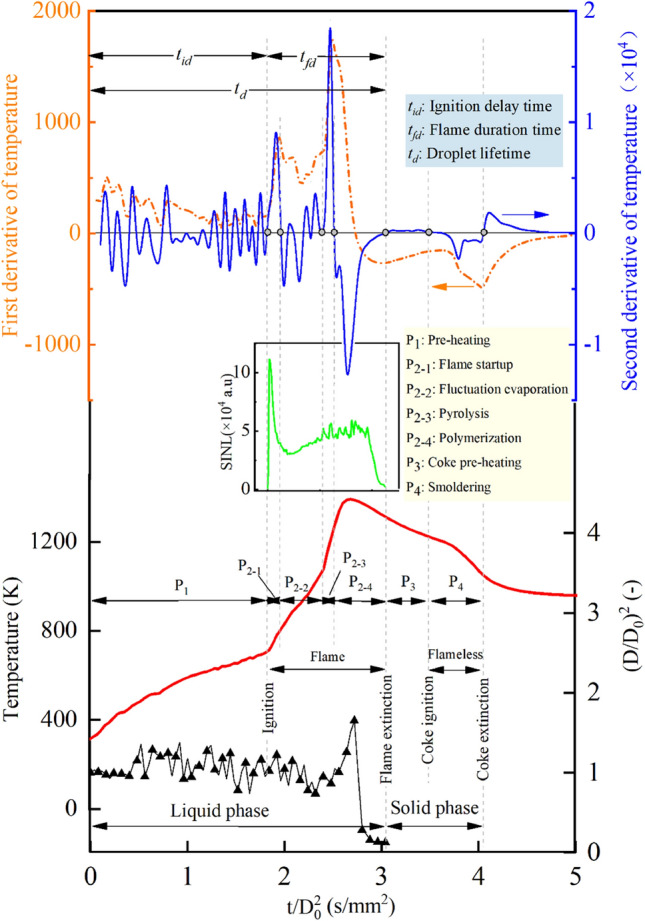
The evolution of droplet temperature, droplet size, the first and second derivative of the temperature of methanol/HFO blend, the SINL curve during flame combustion, and the details of the division of the combustion stage.

Based on the experimental observation, the combustion process of methanol/HFO droplets is identified as a two-phase process involving both liquid and solid phases^36,37^, as depicted in Fig. 2. This two-phase process is further divided into six detailed stages, each of which characterized by notable changes in droplet behavior influenced by various physical and chemical factors. As shown in Fig. 3, it is also noteworthy that the entire combustion process can be divided by the evolution curves of droplet temperature, as well as its first and second-order derivatives. Additionally, Fig. 3 illustrates the variations in SINL throughout the flame combustion period, alongside the progression of the normalized squared diameter. As the combustion progresses, the luminosity of the flame gradually diminishes, and the flame color transitions from pale yellow to tan, and eventually brown. This transformation primarily stems from the formation, agglomeration, and diffusion of soot particles, which results in a gradual accumulation of hydrocarbons involved in combustion.

In the pre-heating stage, methanol and the lighter components of HFO, which distribute on the droplet surface, evaporate and vaporize first, and then diffuse into the ambient environment. As the fuel vapor mixes with air, a combustible mixture is formed. Once the mixture proportion reaches the flammability limits and the vapor temperature increases exceeding the ignition temperature^26^, the droplet will auto-ignite, and a visible flame appears, as shown in Fig. 2. The ignition delay (ID) refers to the time interval between the moment the droplet reaches the observation position in the heating chamber and the moment when a visible flame becomes observable. According to the droplet temperature characteristics curve, the ignition starting point needs to satisfy two conditions: d*T*/d*t* ≥ 0 and d^2^*T/*d*t*^2^ = 0^37^. The ID time recorded by high-speed photography agrees well with the results obtained by the above equations as shown in Fig. 3. This demonstrates the rationality and reliability of using the derivative of the droplet temperature curve to define the characteristic stages.

After the pre-heating stage, the premixed combustion stage follows. In this stage, the combustion of the mixture is characterized by a pale yellow flame that extends from the distal end of the droplet towards its periphery. The combustible mixture burned in this stage is pre-mixed in the pre-heating stage. It indicates the droplet combustion during this stage belongs to premixed combustion. Due to the heating effect of flames on droplets, the slope of the droplet temperature curve during this stage significantly increases, as shown in Fig. 3.

As the combustion process proceeds, the droplet temperature increases continuously. The lighter components in HFO and methanol evaporate preferentially, resulting in a non-uniform distribution of components within the droplet. This evaporation process, driven by component diffusion, leads to an increase in the concentration gradient of heavier components along the radial direction. As a result, an obstructive membrane forms near the droplet’s surface, trapping volatile substances inside. These volatile substances distributed within the droplet act as nucleation sites. When the droplet temperature reaches the corresponding superheat limit temperature (SLT) of the specified component, the nucleation sites are activated, initiating bubbles formation. With increasing droplet temperature as shown in Fig. 3, the volatile substances inside vaporize, causing the bubbles to expand. The growth, movement, coalescence, and eventual collapse of bubbles produce an unstable wave on the droplet surface. When the amplitude of the perturbation wave exceeds the critical thickness of the oil membrane between the bubble and the droplet, the bubble ruptures, and the fuel vapor ejects from the droplet and carries away a portion of the liquid. The frequent puffing caused by bubble rupture is a significant feature of the fluctuation evaporation stage, which starts after the premixed combustion stage and continues until the beginning of pyrolysis.

Figure 3 illustrates the division of the combustion process of methanol/HFO droplets into six stages based on droplet temperature evolution. The inflection points of the temperature trend, indicated by the second derivative reaching zero, are used as the characteristic transition point. This is primarily due to the combustion process being a highly transformative phase with significant temperature fluctuations, and these temperature changes can to some extent indicate the condition of the substance within the droplet. The fluctuation evaporation stage includes two combustion modes: diffusion combustion on the droplet surface and premixed combustion resulting from the combustion of fuel vapor caused by puffing. Due to the presence of oxygen-poor and fuel-rich regions near the droplet surface, diffusion combustion can lead to incomplete oxidation of hydrocarbons, contributing to soot formation. Influenced by natural convection, the agglomeration and diffusion of soot cause a decrease in flame luminosity. Consequently, the color of the flame transitions from pale yellow in the previous stage to tan as shown in Fig. 2. Simultaneously, the flame structure is deformed due to vapor jetting caused by puffing. The combustion of the jetted fuel vapor belongs to the premixed combustion^20^. Figure 3 also illustrates the rate of temperature rise is slower compared to the premixed combustion stage, as some of the absorbed heat is consumed during puffing.

As the combustion process progresses, the evaporation of volatile components during the fluctuation evaporation stage results in an increasing dominance of heavy components. These heavy components, such as asphalt, are colloidal substances with high viscosity. At the end of the fluctuation evaporation stage, the droplets are not entirely in liquid form. As the droplets absorb a substantial amount of heat, the combustion process enters the pyrolysis stage when the droplet temperature reaches the pyrolysis temperature. This stage is characterized by the violent ejection of a substantial quantity of pyrolysis gases, accompanied by the formation of distinct luminous flames in a globular pattern. During this stage, rapid and intense changes in droplet diameter are observed. As most of the lighter components have evaporated, an oil membrane composed of heavier components with high surface tension and viscosity forms, trapping the pyrolysis gases inside the droplet. As the pyrolysis gases accumulate and surpass the surface tension and ambient temperature, vigorous puffing occurs, resulting in the rapid expulsion of pyrolysis gases. Following the rapid restoration of the droplet surface facilitated by the high-viscosity and surface-tension oil membrane, a substantial reduction in the droplet’s diameter occurs. During this stage, intense puffing frequently occurs. The pyrolysis stage terminates at the end of the last maximum intense expansion, signifying the nearly complete release of volatile liquid components in HFO during combustion.

The pyrolysis of heavy components in HFO results in the release of a significant number of gaseous hydrocarbons within the droplet. With the gasification of most heavy components, the viscous membrane rapidly collapses and polymerizes, forming a porous carbonaceous residue known as cenosphere. The cenosphere contains numerous blowholes due to the release of pyrolysis gases^38,39^. As shown in Fig. 3, the slope of droplet temperature in this stage increases, which is primarily attributed to the combustion of the released pyrolysis gas in the pyrolysis stage. Simultaneously, fuel impurities become concentrated, marking the initiation of the polymerization stage. In this stage, aromatic molecular structures predominantly combine to form residual coke^40^. As depicted in Fig. 3, during stage P_2-4_, the droplet size rapidly decreases, and the droplet temperature steadily increases. Once the fuel vapor is depleted, the flame extinguishes. After this stage, the combustion proceeds to the solid phase.

During the solid-phase combustion stage, the coke residual is quickly preheated and ignited with glowing, marking the initiation of the coke-burning process. As heat losses exceed heat generation, the oxidation of the coke residual stops, which leads to the cessation of coke burning^41^. As shown in Fig. 3, the burning of coke causes the temperature measured by the thermocouple to rapidly exceed the temperature of the heating chamber. After the coke is burned, the measured temperature falls back to the ambient temperature. Correspondingly, the feature size of the coke gradually diminishes as the burning process progresses. Although the solid-phase combustion stage accounts for a considerable portion of the overall combustion process in terms of timescale, it generally produces less heat compared to the combustion of the liquid phase. Since the main research focuses on the atomization of liquid fuel in engines, especially the vapor–liquid phase transition, this study primarily centers on the combustion process of liquid-phase droplets until the flame extinguishes, which signifies the lifetime of the droplets.

### Effects of methanol and elevated ambient temperature on combustion characteristics

**Figure 4:**
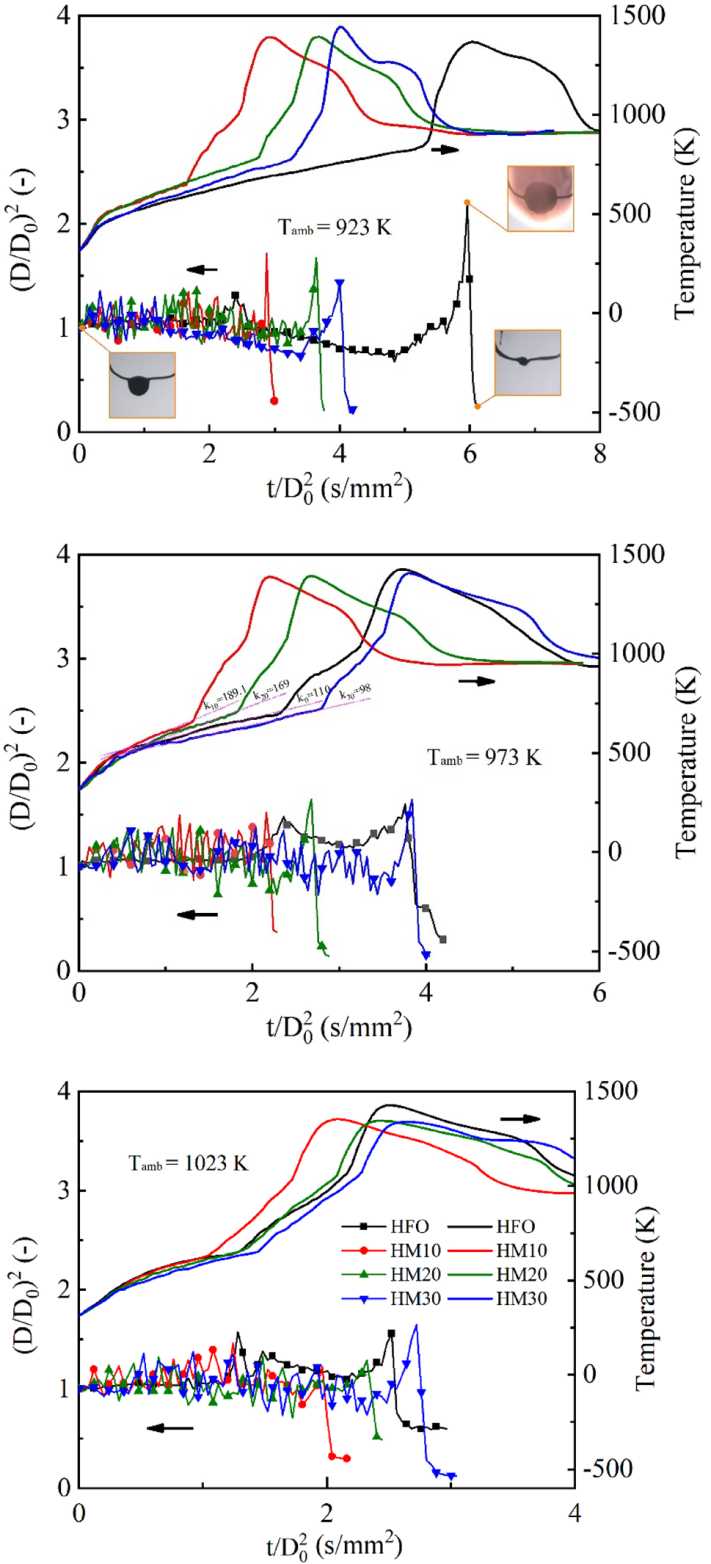
Effect of the ambient temperature and methanol content on combustion characteristics.

Figure 4 shows the droplet’s normalized squared diameter and droplet temperature versus normalized time under various ambient temperatures for HFO, HM10, HM20, and HM30 droplets. It is evident that the behavior of droplet size during combustion is significantly different between HFO and methanol/HFO droplets, particularly in the absence of a distinct fluctuation evaporation stage in HFO. Throughout the entire lifecycle of the droplet, methanol/HFO droplets exhibit pronounced fluctuations in the normalized squared diameter. These fluctuations are primarily driven by the bubbles evaluation, including nucleation, growth, movement, collapse, and rupture, especially the puffing caused by bubble rupture leads to intense fluctuations that respond to the normalized squared diameter, as shown in Fig. 4b. The differences in boiling points of the volatile components in HFO lead to the normalized squared diameter exhibiting strong transient characteristics, which indicates that the specified blend shows distinct morphological characteristics at different times^42^. Additionally, the proportion of light components in HFO is relatively small compared to methanol/HFO droplet. These two factors contribute to the inability of HFO droplets to form large bubbles within a short period of time. As a result, only slight surface oscillation occurs, rather than intense surface fluctuations and droplet deformations. As shown in Fig. 4a, HFO droplets can expand up to 2.2 times their initial size during combustion under an ambient temperature of 923 K. This expansion is attributed to the prolonged ID time, during which the droplet temperature continuously rises before the ignition, transitioning from conventional evaporation to pyrolysis. The extended duration allows for the generation and accumulation of pyrolysis gases, resulting in the expansion of the HFO droplet into a sack-like structure. The temperature evolution curve depicted in Fig. 4 indicates a gradual increase in droplet temperature during the pre-heating stage, followed by a rapid temperature surge due to ignition and intense combustion. For the methanol/HFO droplet as shown in Fig. 4b, the approximate slope of the droplet temperature curve decreases with the increasing methanol content. This can be attributed to the high latent heat of methanol vaporization, and the methanol/HFO droplet with higher methanol content absorbs more heat during evaporation, which slows down the increase in droplet temperature.

**Figure 5:**
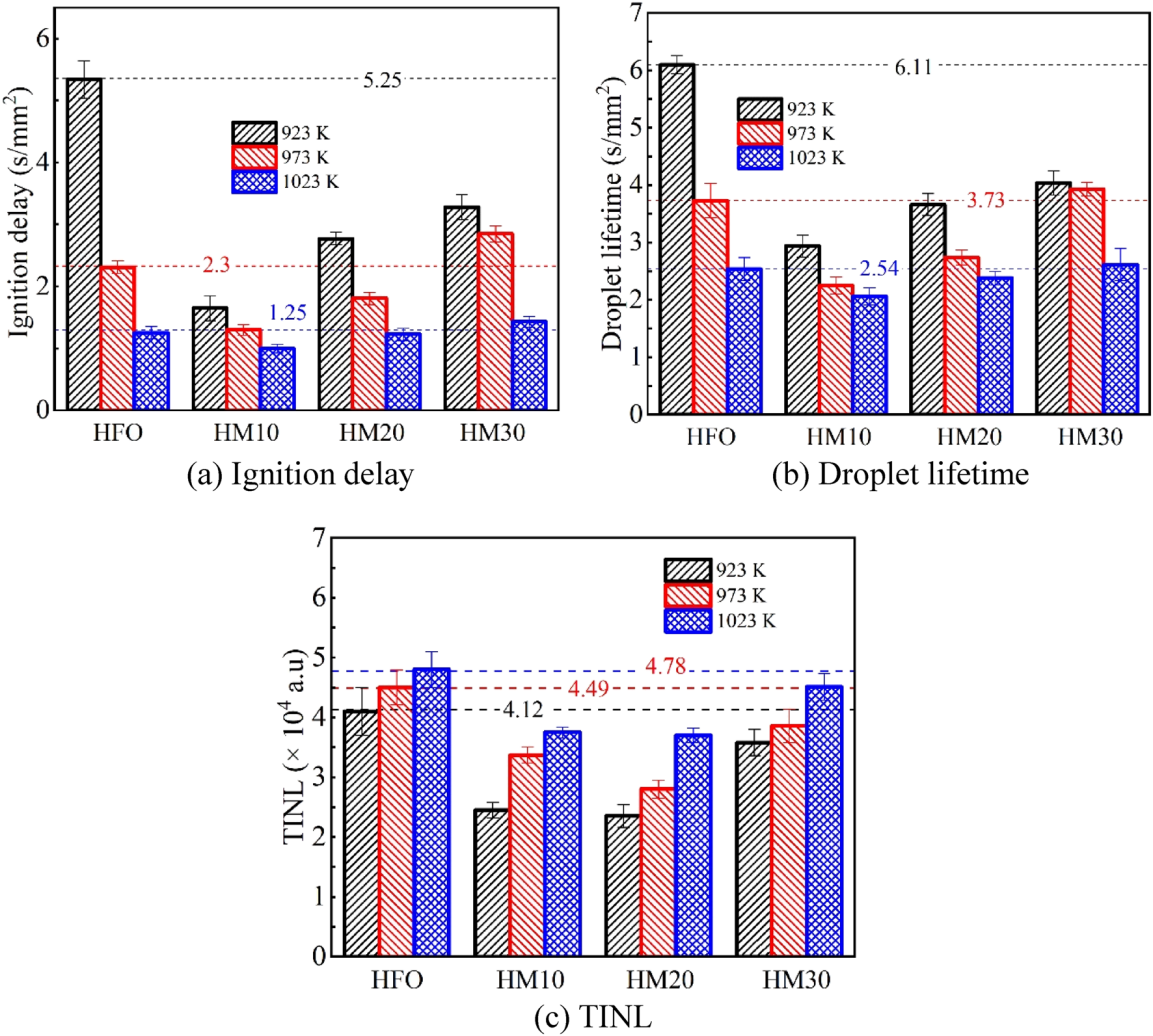
Comparison of the ignition delay, droplet lifetime, and TINL for methanol/HFO droplets with different methanol content at various ambient temperatures.

Figure 5a provides a comparison of the ignition delay for methanol/HFO droplets with different methanol content at various ambient temperatures. The ignition delay can be ascribed to the physical and chemical delay which is generally discussed using the dimensionless number of *D*_*a*_. It is the ratio of the mass transfer characteristic time length *τ*_*m*_ and the chemical reaction characteristic time length *τ*_*c*_. The larger the *D*_*a*_ number the higher the probability of droplet auto-ignition and the shorter ignition delay^43,44^. *D*_*a*_ can be expressed by Eq. (1).

1 $$D_{a} = \frac{{\tau_{m} }}{{\tau_{c} }} = \frac{{(d^{2} /D_{v} )}}{{(\rho /\mathop \omega \limits^{.} )}}$$

2 $$\dot{\omega } = A\rho^{{\alpha_{o} + \alpha_{V} }} (Y_{V} /M_{V} )^{{\alpha_{o} }} (Y_{o} /M_{o} )^{{\alpha_{V} }} \exp ( - E/(RT))$$

where *D*_*v*_ is the diffusion coefficient, $\rho$ is density, $\mathop \omega \limits^{.}$ is chemical reaction rate, A is pre-exponential factor, *Y*, *M*, and $\alpha$ are mass fraction, molecular mass, and index respectively. Their subscripts *V* and *O* represent fuel vapor and oxygen. *E* is the activation energy. According to Ref.^43^, A, *α*_*v*_ and *α*_*o*_ are constant, *Y*_*v*_ and *Y*_*o*_ are nearly uniform at ignition. Therefore, at the fixed initial droplet diameter, Eq. (1) is approximately simplified based on the empirical formula of diffusion coefficients, *D*_*v*_, by Zhang^45^ as follows.

3 $$D_{a} \propto \frac{1}{{T^{2.25} \exp (E/RT)}}$$

Figure 5a shows the increase in ambient temperature leads to a reduction in ignition delay for a fixed fuel. It can be explained by Eq. (3). It indicates that with an increase in ambient temperature, *D*_*a*_ decreases, i.e., the ignition delay shortens. As illustrated by Fig. 5a, at the fixed ambient temperature, for the methanol/HFO blended droplets, the ignition delay prolongs with the increase in methanol content. Due to the significantly lower activation energy of methanol compared to HFO^46,47^, as the methanol content increases, the activation energy, *E*, of the blended fuel, gradually decreases and *D*_*a*_ increases. Based on the experimental observation, different from HFO droplet, frequent and intense puffing occurs after the methanol/HFO droplet is heated, which results from the preferential boiling of methanol. It enhances the airflow disturbance around droplet and accelerates the diffusion of fuel vapor. Thus, the diffusion coefficient, *D*_*v*_, increases, and the mass transfer characteristic time length *τ*_*m*_ decreases. The higher activation energy of HFO compared with methanol/HFO leads to an increase in the chemical reaction characteristic time length *τ*_*c*_. The competition between the two leads to different performances on ignition delay between HFO and methanol/HFO droplets at the same temperature.

As illustrated in Fig. 5b, for the same fuel, the droplet lifetime shows a decrease with increasing ambient temperature. This can be attributed to the accelerated evaporation and combustion processes at higher temperatures, leading to a shorter droplet lifetime. For the methanol/HFO droplet, the droplet lifetime prolongs with the increasing methanol content at the specified ambient temperature. Especially, the droplet lifetime of both HM10 and HM20 is shorter than that of HFO. The main reason is the occurrence of successive puffing, which results in the ejection of secondary droplets during combustion and contributes to sputtering loss in the form of secondary droplet combustion^39^.

Figure 5c illustrates TINL for methanol/HFO droplet with different methanol content at various ambient temperatures. TINL represents the total amount of soot produced throughout the combustion process. The results indicate that all four tested fuels exhibit increased soot production at higher ambient temperatures. This is due to the shortened ignition delay, as higher ambient temperatures exacerbate incomplete hydrocarbon oxidation. The methanol/HFO droplet exhibits reduced TINL compared to HFO at equivalent ambient temperatures, which is consistent with the previous study^48^. Three factors account for this finding. Firstly, during the fluctuation-evaporation combustion stage of the methanol/HFO droplet, the occurrence of periodic puffing intensifies the airflow disturbance around the droplet. This promotes a transition from natural convection to mixed convection including forced convection. As a result, droplet evaporation is enhanced, and fuel vapor diffusion within the flame is improved. Secondly, the combustion of methanol in methanol/HFO droplets generates a substantial amount of OH- radicals, which readily react with soot precursors. This rapid reaction reduces the production of soot during HFO combustion^49^. Thirdly, the evaporation of methanol, characterized by its higher latent heat of vaporization, absorbs a significant amount of heat from the flame. Consequently, lower flame temperatures are achieved. This effect helps to minimize soot formation during combustion^50^. Therefore, the blending of methanol with HFO proves to be an effective approach to reducing soot emissions.

### Analysis of droplet behaviors of methanol/HFO droplet

**Figure 6:**
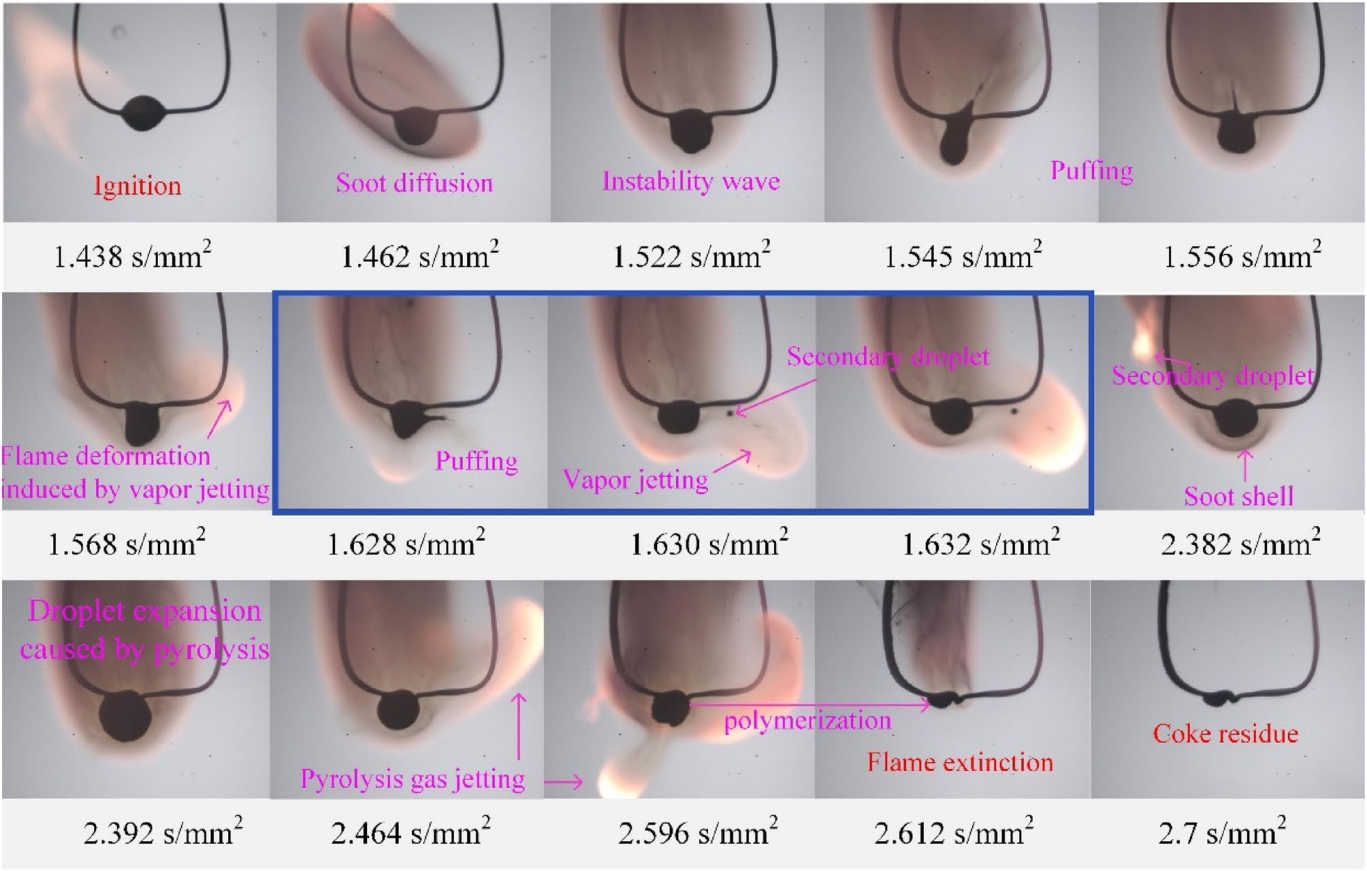
Sequential images during the combustion of methanol/HFO droplets at ambient temperature of 1023 K.

Figure 6 presents a sequence of images depicting the combustion process of methanol/HFO droplets. Following the ignition of droplet, soot is generated and quickly agglomerates, diffusing around the droplet. The preferential boiling of light components and methanol leads to bubble nucleation. Subsequently, bubbles evolve within the droplet, causing density differences at the gas–liquid interface and resulting in surface instability waves, as shown at 1.422 s/mm^2^. Subsequently, the puffing phenomenon appears after the bubble rupture, which results in the formation of liquid ligaments at 1.628 and 1.746 s/mm^2^. The combustion of the jetted fuel vapor presents a luminous flame. The rapid and efficient burning of the fuel vapor outside the droplet induces flame deformation, as observed at 1.768 s/mm^2^. A thick soot shell is observed at 1.802 s/mm^2^, which is formed by incompletely oxidized hydrocarbons during combustion and acts as a barrier hindering the release of burnt gas. The entire puffing process occurs between 1.882 and 1.89 s/mm^2^. The accumulation of pyrolysis gases causes the droplet to expand to its maximum size at 2.382 s/mm^2^. Puffing, accompanied by the rapid release of pyrolysis gases, and a luminous flame, is observed at 2.394 and 2.396 s/mm^2^ in Fig. 6. Finally, as the volatile components are depleted, the flame extinguishes at 2.4 s/mm^2^. Finally, as the volatile components are depleted, the flame extinguishes at 2.4 s/mm^2^.

**Figure 7:**
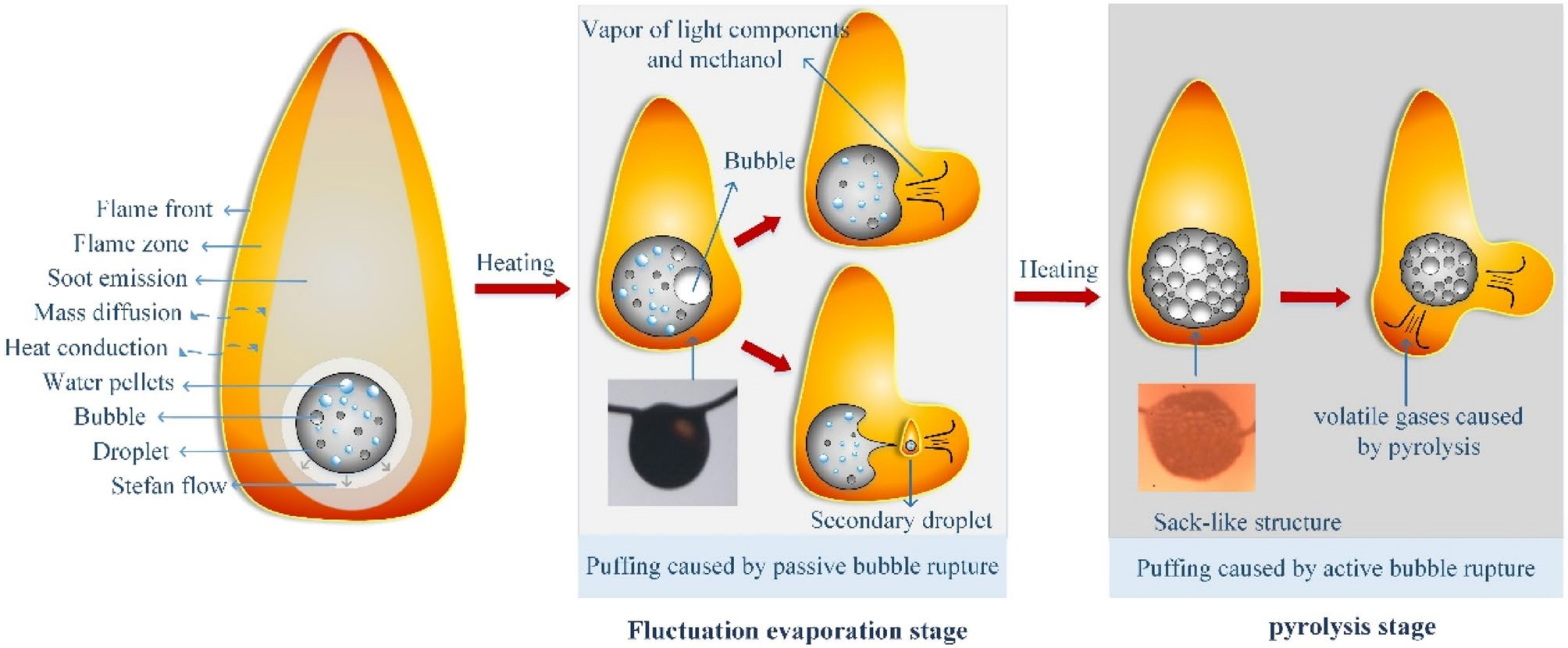
Diagram of droplet flame structure, and the puffing behaviors during the stages of fluctuation evaporation and pyrolysis.

Based on experimental observations, the droplet flame structure and two types of puffing phenomena during the combustion of HM30 droplets are summarized and illustrated in Fig. 7. It is evident that puffing is primarily caused by bubble rupture. Based on the mechanism, bubble rupture is classified into active and passive types, which occur respectively during the fluctuation evaporation stage and pyrolysis combustion stage. During the fluctuation evaporation stage, the superheated methanol within the droplet nucleates and grows sequentially. The rapid internal boiling of multiple bubbles and the density difference across the gas–liquid interface induces Rayleigh–Taylor (RT) and Landau-Darrieus (LD) instability waves on the droplet surface^51^, as shown in 1.522 s/mm^2^ in Fig. 6. Bubble rupture occurs when the amplitude of instability waves exceeds the thickness of the oil membrane. This type of bubble rupture caused by the disturbances of instability waves is defined as passive rupture.

After the occurrence of passive bubble ruptures, a conical cavity is formed on the droplet surface, and the ligament gradually extends from the center of the cavity. The evolution of the ligament is mainly controlled by the surface tension and ligament inertia force. Previous reports have indicated that the size of the bubble determines the formation of ligaments^52^. For small bubbles, their rupture may only result in oscillation on the droplet surface, as shown in the image at 1.568 s/mm^2^ in Fig. 6. However, the rupture of a large bubble leads to a violent re-equilibrium of the cavity, causing the development of an outward-pushing ligament. Under the influence of Plateau-Rayleigh instability^52^, the ligament eventually pinches off and breaks up, resulting in the separation of the secondary droplet from the primary droplet at the ligament tip, as shown in the images at 1.545, 1.556, and 1.628 s/mm^2^ in Fig. 6. Simultaneously, the vapor inside the bubble is ejected and undergoes combustion, leading to the generation of a luminous flame and causing deformation in the flame shape.

During the pyrolysis stage of droplet combustion, the oil membrane transforms into a high-viscosity and high-surface tension shell. This transformation causes the membrane to behave more like a solid substance, significantly limiting the generation of instability waves. It is widely understood that the pyrolysis of the heavy components in HFO, such as asphalt, results in the release of gaseous hydrocarbons and the formation of a carbonaceous residue^38^. Puffing in this stage primarily occurs due to the accumulation of pyrolysis gases, leading to a continuous increase in vapor content and pressure within the bubbles. When the vapor pressure exceeds the combined effect of surface tension and atmospheric pressure, active bubble rupture occurs, resulting in the rapid ejection of pyrolysis gases. Due to the high viscosity and high surface tension of the oil membrane during this stage, vapor jetting does not generate intense instability waves on the droplet surface. The cavity formed after vapor jetting quickly collapses and contracts due to the constraints imposed by the dense oil membrane. Puffing during the pyrolysis stage is characterized by the absence of ligament formation and secondary droplet generation. The vapor inside the bubble during this stage consists mainly of pyrolysis gases. Although these two types of puffing exhibited different bubble rupture forms, the flame instability is intensified significantly in both cases.

**Figure 8:**
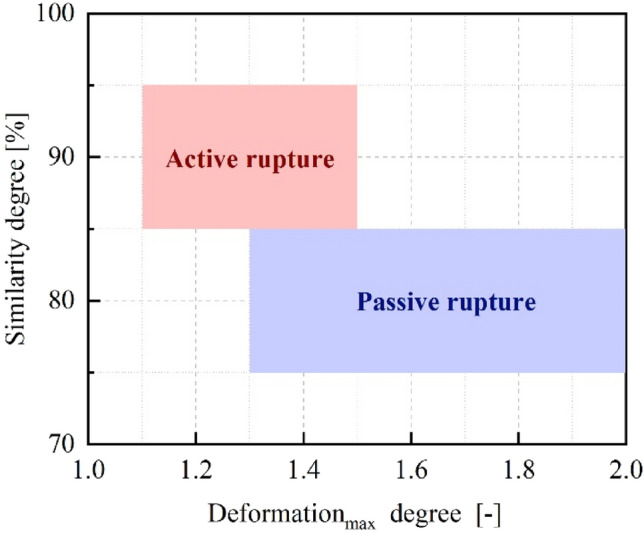
The classification criteria of passive and active rupture of bubbles.

The breakup of the superheated blended fuel droplets can occur in two patterns: micro-explosion and puffing^53^. In this study, only puffing was observed. To quantitatively analyze the puffing process, the classification criteria were established based on *Similarity* and *Deformation*_*max*_ encompassing both puffings triggered by the passive and active rupture of bubbles^54^.

4 $$Similarity = 100 - \sqrt {\frac{{\sum\limits_{i,j}^{500 \times 500} {(P_{m} (i,j) - P_{n} (i,j))^{2} } }}{500 \times 500}}$$

5 $$Deformation_{\max } = \frac{{S_{c} }}{S} = \frac{{C^{2} }}{4\pi S}$$

where *P*_*m*_(*i,* *j*) represents the logical value 0 or 1 at the pixel point (*i,* *j*) before puffing, and *P*_*n*_ (*i,* *j*) denotes the logical value 0 or 1 at the pixel point (*i,* *j*) after puffing. Similarity reflects the difference between the two images before and after puffing. *S*_*c*_ represents the equivalent standard circle area derived from the droplet projection’s perimeter. *C* and *S* are the perimeter and area of the actual droplet projection. Deformation_max_ characterizes the maximum deformation degree during the puffing process. As indicated in Fig. 8, when the similarity degree is between 85 and 95%, and the maximum deformation degree is between 1.1 and 1.5, puffing is primarily caused by active rupture. When the similarity degree is between 75 and 85%, and the maximum deformation degree is between 1.3 and 2.0, puffing is primarily caused by passive rupture.

## Conclusion

The combustion of methanol/HFO droplets with a methanol content from 10 to 30% was experimentally investigated using the suspended droplet method under ambient temperatures of 923, 973, and 1023 K. The combustion characteristics of the methanol/HFO droplet were analyzed, and the main conclusions were summarized as follows:

1. The combustion of methanol/HFO droplets was classified into six stages, primarily emphasizing liquid-phase combustion, including pre-heating, premixed combustion, fluctuation evaporation, pyrolysis, and polymerization stage. Fluctuation evaporation was identified as a distinctive characteristic exhibited by methanol/HFO droplets during combustion.
2. The effects of ambient temperature and methanol content on characteristics curves of normalized squared diameter and temperature of HFO, HM10, HM20, and HM30 droplets during combustion were compared and analyzed. The fluctuation evaporation stage was found to be a distinct phenomenon in the combustion process of methanol/HFO droplets, setting it apart from HFO.
3. Both the ignition delay and droplet lifetime of HFO and methanol/HFO droplets decreased with increasing ambient temperature. At the specified ambient temperature, for the methanol/HFO droplet, the ignition delay and droplet lifetime increased with the increasing methanol content. The TINL was lower for the methanol/HFO blend compared to HFO. Prominently, HM10 had the most significant reduction in droplet lifetime and TINL compared to HFO, which indicated that the addition of 10% methanol accelerated the combustion process and reduced the soot generation, making the blended fuel a promising alternative fuel to realize high-efficiency combustion for marine diesel engine.
4. Based on the analysis of thermos-dynamic characteristics of methanol/HFO droplets during combustion, it was found that puffing was primarily attributed to the superheating of methanol and the pyrolysis of heavy components in HFO, which resulted in active and passive rupture of bubbles respectively. Similarity and Deformation_max_ were employed to qualitatively distinguish between active and passive rupture of bubbles.

## Data Availability

All data generated or analyzed during this study are included in this published article.
